# Do Behavioral Risk Factors for Prediabetes and Insulin Resistance Differ across the Socioeconomic Gradient? Results from a Community-Based Epidemiologic Survey

**DOI:** 10.1155/2015/806257

**Published:** 2015-05-19

**Authors:** May H. Yang, Sue A. Hall, Rebecca S. Piccolo, Nancy N. Maserejian, John B. McKinlay

**Affiliations:** ^1^New England Research Institutes, Inc., Watertown, MA 02472, USA; ^2^Department of Epidemiology, New England Research Institutes, Inc., 480 Pleasant Street, Suite 100A, Watertown, MA 02472, USA

## Abstract

To examine whether behavioral risk factors associated with diabetes (diet, BMI, waist circumference, physical activity, and sleep duration) are also related to both prediabetes and insulin resistance (IR), we used data from Boston Area Community Health (BACH) Survey (2010–2012, *n* = 3155). Logistic and linear regression models were used to test the association of lifestyle factors with prediabetes status, insulin resistance, and prediabetes or insulin resistance. All regression models were stratified by education and income levels (to examine whether risk factors had differential effects across socioeconomic factors) and adjusted for age, gender, race/ethnicity, family history of diabetes, and smoking status. We found that large waist circumference was consistently associated with higher levels of insulin resistance (IR) and increased odds of prediabetes. While the association between large waist circumference and IR was consistent across all levels of SES (*P* < 0.001), the association between large waist circumference and prediabetes was only statistically significant in the highest socioeconomic strata with odds ratios of 1.68 (95% CI 1.07–2.62) and 1.88 (95% CI 1.22–2.92) for postgraduate degree and income strata, respectively. There was no association between diet, physical activity, sleep duration, and the presence of multiple risk factors and prediabetes or IR within SES strata.

## 1. Introduction

The prevalence of type 2 diabetes continues to rise and currently affects over 25 million Americans and is estimated to reach 439 million adults worldwide by 2030 [1, 2]. Along with this increase, the prevalence of prediabetes is also rising, affecting approximately one in three U.S. adults aged ≥ 20 years (an estimated 79 million individuals) [3, 4]. Furthermore, it is estimated that, among U.S. adults with undiagnosed diabetes or impaired fasting glucose, the proportion of adults with insulin resistance increased from 24.8% to 31.1% during the time periods of 1988–1994 to 1999–2002 [5].

Prediabetes is indicated when blood glucose or hemoglobin A1c (A1c) levels are higher than normal, but not yet high enough to be classified as diabetes. Prediabetes raises the risk of type 2 diabetes by 3- to 10-fold and it is estimated that up to 70% of people with prediabetes may develop type 2 diabetes during their lifetime [3, 6, 7]. A study conducted by Geiss et al. [8] estimates 30% of the U.S. adult population had prediabetes in 2005-2006, but only 7% were aware that they had the condition. Insulin resistance (IR) is a condition in which the body produces insulin but does not use it effectively. When people have insulin resistance, glucose builds up in the blood instead of being absorbed by the cells, leading to type 2 diabetes or prediabetes.

The American Diabetes Association (ADA) (April 4) [9] considers both prediabetes and insulin resistance precursors to type 2 diabetes. Both the ADA and the National Heart Lung and Blood Institute's National Cholesterol Education Program (NCEP) [10] suggest the risk factors for type 2 diabetes, prediabetes, and IR are similar and include the following (not comprehensive): obesity, physical inactivity, family history of diabetes, race/ethnicity, high blood pressure, large waist circumference, and high triglycerides. Notably, socioeconomic status is usually not included but is an important focus of this paper.

Socioeconomic status is a complex construct, broadly based on relative income, education, and occupation—all of which have been repeatedly associated with lifestyles and behavioral risk factors. In this analysis, we use income and education as indicators of SES, since occupation has been shown to be highly correlated with education [11, 12]. Numerous studies have identified low physical activity [13–15], low diet quality [14, 16, 17], and, more recently, poor sleep quality, as associated with diabetes [18–22]. However, it is uncertain whether these factors equally affect prediabetes and diabetes risk across the SES gradient. That is, most previous studies statistically adjust for education, income, or some other marker of SES [23–25]. But to develop well-targeted and likely effective primary and secondary interventions it is necessary to understand how modifiable risk factors influence risk of diabetes among the different SES groups. Studies have shown that while health programs and therapies exist to manage prevention of diabetes and its complications, these programs are underutilized among those in low socioeconomic groups [26–28].

While there are many risk factors contributing to diabetes, this study seeks to identify those most likely to improve the effectiveness of primary and secondary prevention initiatives. Coupled with the under-utilization of diabetes prevention programs among those with low SES [26–28], there is a potential benefit to identifying modifiable diabetes risk factors that may contribute to prediabetes among those with low SES and to examine the different effects of diabetes risk factors operating among different SES levels.

Therefore, the objectives of this analysis are

1. within and across each level of SES, to examine the associations between known behavioral risk factors for diabetes (low diet quality, poor sleep, low physical activity, and high waist circumference) and both prediabetes and IR;
2. to examine these modifiable factors collectively (presence of two or more risk factors) for their potential impact on the prevalence of prediabetes and IR within and across the SES gradient.

## 2. Materials and Methods

### 2.1. Participants

The Boston Area Community Health (BACH) Survey is a longitudinal cohort study of residents of Boston, MA, aged 30–79 years at baseline (March 2002–June 2005). Detailed methods have been described elsewhere [29, 30]. Briefly, a stratified two-stage cluster sample was used to recruit an approximately equal number of participants by gender, race/ethnicity (Black, Hispanic, White), and age group (30–39, 40–49, 50–59, 60–79). 5,502 adults participated in baseline BACH I (1767 Black, 1876 Hispanic, 1859 White; 2301 men, 3201 women). Follow-up surveys were collected at two time points approximately 5 (BACH II 2006–2010) and 7 (BACH III 2010–2012) years later. For BACH III, completed interviews were obtained for 3155 individuals (1184 men; 1971 women). In all surveys, data were collected during a two-hour interview in English or Spanish, after obtaining written informed consent. The study was approved by New England Research Institutes' Institutional Review Board. Analyses for this paper use data from the most recent interview, BACH III (2010–2012).

### 2.2. Sociodemographic Lifestyle Assessment Measures

Education, based on years of education completed, is composed of four categories: <high school, high school or GED, some college, or college or advanced degree. Income was collected as total annual income and categorized into three categories: <$20,000, $20,000–$49,999, or $50,000+. Physical Activity was measured using the Physical Activity for the Elderly (PASE) scale and categorized into low, moderate, or high [31]. Anthropometric measures-Trained field Interviewers directly measured the subject's height (cm), weight (kg), from which BMI was calculated (kg/m^2^), and waist circumference (cm). Waist circumference was further dichotomized into high waist circumference defined as ≥88 cm for women and ≥102 cm for men [32, 33]. BMI was dichotomized using the cut-off point of 25 kg/m^2^ that defines overweight in the current WHO classification.

Diet was measured with the Block Food Frequency Questionnaire (FFQ) administered in English or Spanish. The FFQ has been validated to obtain data on usual dietary intake over the past year [34]. We calculated an overall healthy eating score using the USDA and AHA guidelines for healthy eating [35, 36]. The healthy eating score is composed of FFQ data on average daily intake of sodium (g), vegetables (servings/day), fruits (servings/day), meats/beans (serving/day), grains (servings/day), fiber (g), and saturated fat (g). Participants were classified into three possible diet score groupings: poor (score 0–2 points), intermediate (score 2–5 points), or high (score 5–7 points). The overall healthy eating index for this sample ranged from 0 to 5 indicating that no one met criteria for high (healthiest) healthy eating score. Therefore, for analyses, we created 3 groups (low/medium/high) based on off tertile cut-points. Each individual component of the diet score was also examined separately.

Sleep duration was measured using self-reported sleep patterns in the previous month with the question “How many hours of actual sleep do you get during the night?” and grouped as follows: <6.5 hours, 6.5–<8 hours, and ≥8 hours to create low, intermediate, and high duration of sleep.

Multiple risk factors measures were created by combining diet, sleep, and activity scores. The presence of two factors is defined as having poor diet (lowest tertile) and low sleep duration (<6.5 hours). The three-risk-factor measure is defined as the presence of low physical activity, poor diet, and low sleep duration. Lastly the four-risk-factor measure is defined as the presence of high BMI (≥25) or high waist circumference (≥88 cm women and ≥102 cm men) and presence of the three risk factors mentioned above.

### 2.3. Major Outcome Measures

Diagnosed type 2 diabetes was determined by self-report (Have you ever been told by a doctor or other health professional that you have type 2 diabetes?). Medication inventory confirmed over 80% of the self-reported cases of diabetes. Insulin resistance was measured using the homeostasis model assessment (HOMA) index where the product of fasting glucose and fasting insulin was taken and then divided by 405 [37]. Insulin resistance was calculated only in subjects that had fasting glucose collected and has an analytic sample size of 2359. Prediabetes is defined among those subjects with no diabetes (type 1 or type 2) and no undiagnosed diabetes defined as fasting glucose >125 or HbA1c ≥6.5. Subjects were considered prediabetic if they have fasting glucose 100–125 or HbA1c 5.7–6.4 [38]. Subjects with prediabetes are compared to diabetes unaffected subjects—that is, those without diabetes (self-reported type 1 or 2 or undiagnosed) and with no prediabetes leaving an analytic sample size of 2175. A third outcome combining prediabetes and insulin resistance was created to capture the presence of prediabetes or insulin resistance among nondiabetic subjects (*N* = 2175). To define insulin resistance, we used the NHANES study cut-point of ≥2.73 [39].

### 2.4. Statistical Analysis

Bivariate associations between health factors and education were examined with Wald F Chi-Square tests for categorical measures and Wald F test *P* value from linear regression models for continuous measures.

Logistic regression models were utilized to examine the association of lifestyle/behavioral health factors with prediabetes and the combined outcome of insulin resistance or prediabetes. Linear regression models were constructed to examine the association between lifestyle and behavioral factors on insulin resistance. Due to positive skew, insulin resistance was log transformed in final regressions. In all regression models we stratified by education level or income group and adjusted for age, gender, race/ethnicity, family history of diabetes, and smoking status. Regression models were examined for the overall healthy eating score as well as each of the 7 subscales but results are presented only for the overall score. Analyses of diet were adjusted for total caloric intake to minimize measurement error by over/underreporting [40]. Separate unstratified regression models were examined to test for interactions between lifestyle and behavioral factors and SES measures income and education.

All analyses were performed with SAS callable SUDAAN 11.0 [41] using sampling weights and stratification measures to account the complex survey design of BACH. Multiple imputation was used to reduce bias resulting from missing data for all exposure covariates using multivariate imputation by chained equations (MICE) in R [3]. Briefly, MICE imputes missing values with estimated predictions from regression models that reflect the relationships observed in the data, while considering the complex survey sampling design. Age, race, and their interaction were included as predictors in each of the imputation models. In addition, mice selected important predictors for each variable and these were also included in the model. Less than 5% of the covariates (age, gender, education, race/ethnicity, and income) were imputed [42]. Fifteen multiple imputation datasets were created stratified by race/ethnicity by gender.

## 3. Results and Discussion

The study population characteristics are shown in Table 1. Among the total sample, 28% had normal blood glucose levels (without self-reported diabetes and not prediabetic). In the sample without diabetes (*n* = 2379) 65% were prediabetic and 69% were prediabetic or IR. The geometric mean of the HOMA-IR measure was 1.9 (SE 1.0). Among those with prediabetes, over half were White and reported medium levels of physical activity and 46% reported a family history of diabetes. In subjects with prediabetes or IR, 45% were male and reported a family history of diabetes. The mean waist circumference for those with prediabetes or IR is the highest at 96 cm compared to those unaffected or with prediabetes alone. In general, mean diet FFQ scores (total score and subscales) were comparable across groups.

Similar patterns were observed in the bivariate associations between study population characteristics with educational level and income (data not shown). Educational level is significantly associated with most measures except for total FFQ score. Males were more likely to have higher education (postgraduate) compared to female (51% versus 49%, *P* = 0.04). The <HS group also reported the highest rates of current smoking and family history of diabetes compared to all other education levels (*P* ≤ 0.0001 in all comparisons). Regarding diabetes outcomes, subjects in the highest education group had higher rates of being in the unaffected group (34% versus 12%, *P* ≤ 0.0001) and lower rates of reporting prediabetes (61% versus 80%, *P* ≤ 0.0001) and prediabetes or IR (65% versus 83%, *P* = 0.006) compared to the lowest education group. Gender, current smoking, family history and diabetes, and the diabetes outcomes were associated with income with similar patterns.

**Figure 1:**
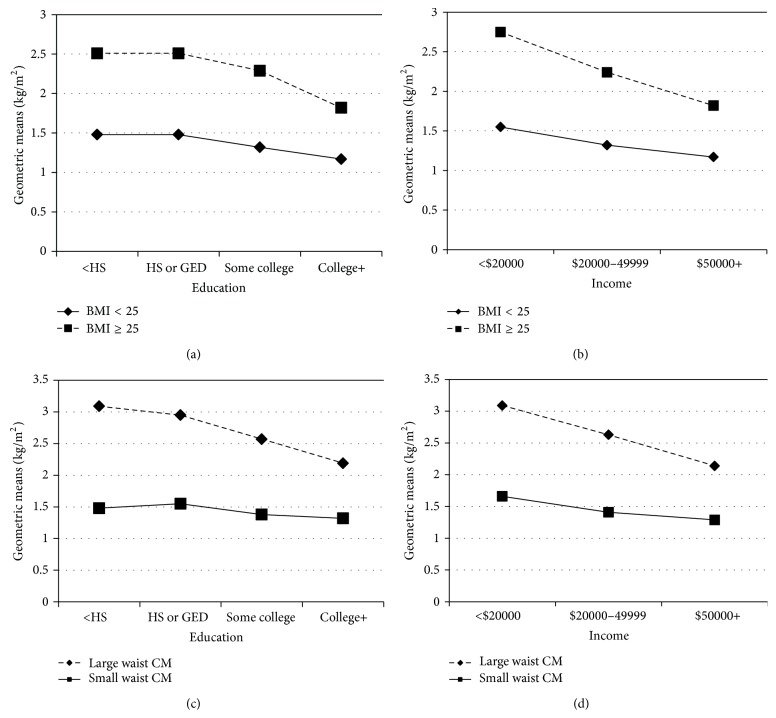
Geometric means of BMI and waist circumference by education and income strata.

In unstratified models there were no significant interactions seen between education and income with lifestyle/behavioral factors (data not shown).  Table 2 displays the results of the insulin resistance regressions and Figure 1 plots mean HOMA-IR for significant predictors. Insulin resistance is associated with BMI and waist circumference across all levels of education and income (*P* ranges from 0.02 to ≤0.0001, Table 2, Models 2 and 3). The magnitudes of association with BMI and waist circumference on IR are similar across all levels of education and income. Those with BMI ≥ 25 have on average a higher mean log IR score of 0.20 compared to those with BMI < 25. Similarly, those with a high waist circumference have higher mean IR scores anywhere between 0.22 and 0.33 points higher than those with a smaller waist circumference. The healthy eating score, other diet components, physical activity, and sleep duration were not associated with insulin resistance. In addition, presence of multiple risk factors was not predictive of insulin resistance.

The covariate adjusted geometric means for insulin resistance in the BMI ≥ 25 group are nearly twice as high compared to the BMI < 25 group within the lowest education level <HS or GED (Figure 1). The same magnitude in mean differences between the BMI categories is also seen in the HS or GED and some college group. Geometric HOMA-IR means are also higher in those with BMI ≥ 25 compared to BMI < 25 within income levels. Across both education and income groups, HOMA-IR geometric means are lower as one moves into higher educated or income groups. The geometric means for those with BMI ≥ 25 are 2.8, 2.2, and 1.8 for the income groups <$20,000, $20,000–$49,000, and $50,000+, respectively. With regard to waist circumference, higher mean HOMA-IR values are observed among subjects with large waist cm compared to small waist cm within all levels of education and income (*P* ≤ 0.0001 in all models). These differences are nearly threefold.

Few significant associations were seen in logistic regression models of prediabetes (Table 3). Large waist circumference was significantly associated with increased odds of prediabetes in the college or advance degree group such that those with large waist circumference were 1.7 times as likely to develop prediabetes compared to those with lower waist circumference (OR 1.68, 95% CI: 1.07–2.62). Similarly for income, subjects with large waist cm were nearly twice as likely to have prediabetes compared to those of lower waist cm in the medium and high income strata with OR 2.13 (95% CI: 1.10–4.10), and OR 1.88 (95% CI: 1.22–2.92) for medium and high income respectively. As with insulin resistance, diet, physical activity, sleep duration, and the presence of multiple risk factors were not associated with prediabetes.

**Figure 2:**
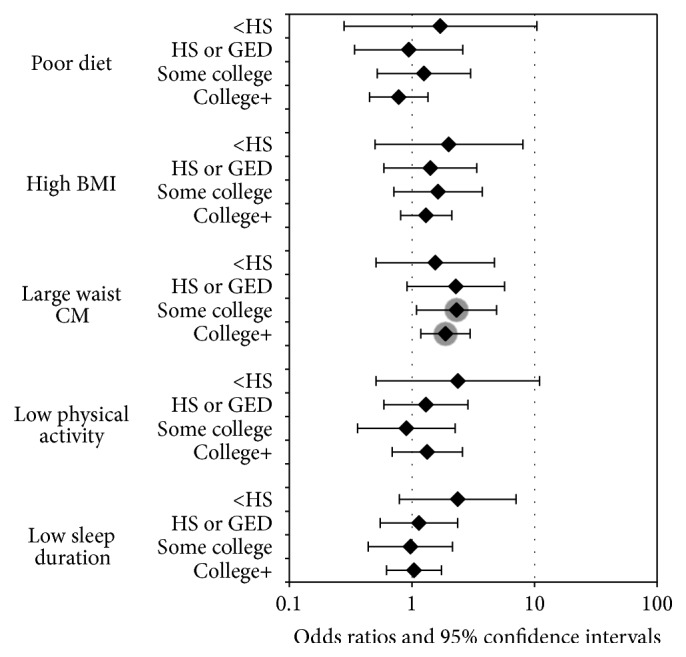
Odds ratio estimates for prediabetes or insulin resistance by education strata.

**Figure 3:**
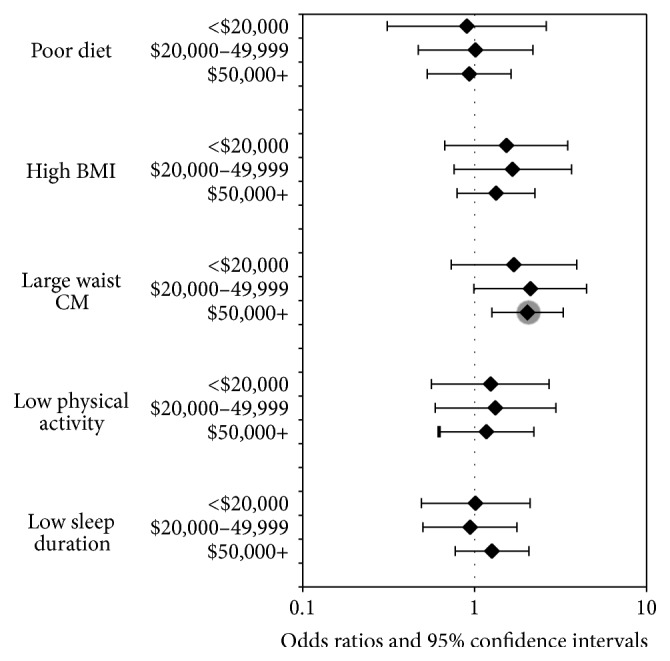
Odds ratio estimates for prediabetes or insulin resistance by income strata.

Figures 2 and 3 display the results of the prediabetes or IR logistic regression models for strata of education and income, respectively. We see similar patterns to the insulin resistance and prediabetes models. Waist circumference remained a significant predictor of prediabetes or IR. Subjects with a large waist circumference were nearly twice as likely to develop prediabetes or IR in the higher education strata (some college and college+) and among the highest income group ($50,000+). The association with multiple risk factors remained nonsignificant. The odds ratios for large waist circumference are in the same positive direction and of similar magnitude across all education levels with a range of 1.55 to 1.88, where a larger waist circumference nearly doubles the likelihood of prediabetes or IR. However the association in the <HS and HS or GED strata is nonsignificant. The same strength of association with waist circumference is seen between income strata, where the odds ratios range between 1.69 and 2.11 and reached significance only within the highest income strata.

## 4. Conclusions

Our analysis shows that, after adjusting for SES indicators (education and income), commonly known diabetes behavioral risk factors (namely, diet quality, sleep duration, and physical activity) are poor predictors of prediabetes and insulin resistance. Larger waist circumference was a consistent predictor of prediabetes and insulin resistance with large waist circumference increasing the probability of prediabetes or IR nearly twofold. While this magnitude of effect is seen across all levels of SES, it reaches significance primarily within higher SES levels. The examination of multiple risk factors revealed that the presence of two or more risk factors is not associated with prediabetes or IR. While many studies have shown significant associations between diet and diabetes risk [17, 27, 43–45], these results have been mixed. For example, other studies have shown no association between diet quality, specifically total fat intake and red meat are not predictive of diabetes risk [46, 47]. Other studies have shown that BMI adjustment attenuates the association between diet quality and type 2 diabetes [48–50] which supports our findings of BMI being a significant predictor of insulin resistance across all levels of SES.

The significant findings between BMI and waist circumference indicate that, independent of education and income, increased BMI and waist circumference are important predictors of prediabetes and insulin resistance. The strength of our waist circumference results is corroborated by a study which examined the interrelationships between demographic (age, income, marital, race, and education) and physical activity and poor diet on prediabetes in path models, concluding that large waist circumference had the strongest direct effect on prediabetes [51]. In addition, findings from the NHANES 2009-2010 survey indicated that the significance of sleep disorders on diabetes is attenuated when BMI is added to the model, where the odds ratio for diabetes drops from 2.04 (1.40,2.95) to 1.38 (0.95,2.00). They conclude that the effect of sleep disorders on diabetes may be explained through a subject's obesity status [52]. While they controlled for various factors such as age, gender, ethnicity, education, and income, they did not examine other modifiable risk factors (e.g., diet and sleep).

The significant association of waist circumference with both prediabetes and insulin resistance may help guide future primary and secondary prevention programs where, in addition to socioeconomic factors, subjects with high BMI and large waist circumference are likely to produce the most beneficial outcomes. The finding that waist circumference is not predictive across all education levels in prediabetes suggests that there may be different predictors of prediabetes among the lower SES groups providing new opportunities for more targeted intervention programs. On a broader level, our analyses showed no association between known diabetes risk factors and prediabetes. This may be explained by the fact that prediabetes is considered an early precursor state indicating likely eventual development of diabetes, and not the eventual established diabetes state itself, by which time the associated risk factors are more evident and strongly associated. Moreover, while the majority of prediabetic cases may end up being diagnosed with diabetes, not all cases will be; therefore the expected relationship of known diabetes risk factors with prediabetes is diluted.

There are some limitations to this epidemiologic investigation. First, because it is cross-sectional; the temporality of the relationships uncovered remains uncertain. Fasting insulin was also only measured at a single time point and maybe subject to measurement error. The fact that measures are obtained at a single time point may reduce the likelihood of finding significant differences as research has shown that repeated measures of health behaviors may increase the significance and the effect size of such modifiable behaviors [53]. Physical, direct measures such as BMI and waist circumference may serve as a lifetime proxy for diet quality and level of physical activity compared to self-reported measures of health behaviors, taken at a single time point. While the reliability of self-reports of health conditions may be questioned, there is evidence that they are generally well-correlated with medical record review [54–57]. Fortunately, medication data were available and over 80% of those self-reporting diabetes were taking a diabetes-related medication.

Countervailing strengths of this study include use of a community-based random sample and the composition of the sample, which covers a broad age range, inclusion of both genders, and a racial/ethnic diversity, with roughly equal numbers of Blacks, Whites, and Hispanic participants. Inclusion of a broad range of recognized behavioral risk factors permitted assessment of their independent and joint influences on prediabetic states. Finally, we examined a variety and combination of multiple risk factors, sleep duration, diet, and physical activity to test whether targeting multiple behaviors were predictive of prediabetes or IR. We found that, among the behavioral risk factors considered, BMI and waist circumference were consistent predictors of prediabetes outcomes independent of SES. Our results have both clinical and public health significance: many different risk factors (including BMI and waist circumference) are variably associated with diabetes, prediabetes, and IR and offer variably effective opportunities for primary and secondary prevention. By identifying, among the broad range of risk factors, the most promising influences, future primary and secondary prevention initiatives can be more precisely targeted, resulting in more effective and cost efficient outcomes.

## Figures and Tables

**Table 1:** Characteristics of BACH-3 analytic sample (*N* = 3155), overall and by fasting glucose/diabetes status.

	Total *N* = 3155	Fasting glucose/diabetes status			
		Unaffected *N* = 641 (27.7%)	Prediabetes^***^ *N* = 1533 (64.6%)	Prediabetes or insulin resistant *N* = 1627 (69.2%)^***^	Insulin resistance HOMA model^*^ *N* = 2359 Mean (SE) = 1.9 (1.0)
Age, y mean SE	54.0 (0.5)	50.8 (0.8)	53.7 (0.6)	53.5 (0.6)	0.05
Gender, %					
Male	1184 (46.5%)	241 (47.8%)	559 (43.9%)	599 (44.5%)	2.04 (1.05)
Female	1971 (53.5%)	399 (52.2%)	973 (56.1%)	1028 (55.5%)	1.73 (1.05)
BMI, mean SE	29.5 (0.2)	27.6 (0.3)	29.3 (0.3)	29.4 (0.3%)	0.41
Waist circumference, cm mean SE	97.0 (0.5)	91.9 (1.0)	95.8 (0.7)	96.2 (0.6%)	0.45
Education					
<HS	618 (7.9%)	76 (3.4%)	246 (7.2%)	259 (7.0%)	2.24 (1.09)
HS or GED	948 (24.4%)	173 (21%)	454 (23.1%)	483 (23.1%)	2.24 (1.06)
Some college	671 (20.5%)	139 (18.2%)	332 (21%)	354 (21.2%)	2.06 (1.07)
College or advanced	916 (47.1%)	251 (57.4%)	499 (48.7%)	531 (48.8%)	1.60 (1.05)
Income					
<$20,000	1338 (27.0%)	204 (19.1%)	577 (23.8%)	615 (24.5%)	2.43 (1.07)
$20,000–$49,999	914 (25.1%)	181 (21.2%)	472 (27.1%)	498 (26.7%)	1.97 (1.05)
>$50,000	902 (48.0%)	93 (59.7%)	482 (49.2%)	514 (48.8%)	1.68 (1.07)
Race/ethnicity					
Black	1026 (27.1%)	140 (13.8%)	504 (29.1%)	531 (28.6%)	2.30 (1.06)
Hispanic	1036 (12.2%)	195 (11.6%)	475 (12.5%)	507 (12.7%)	1.96 (1.08)
White	1093 (60.7%)	305 (74.6%)	553 (58.4%)	589 (58.7%)	1.69 (1.05)
Physical activity					
Low	1244 (32.2%)	185 (25%)	546 (30.7%)	580 (30.8%)	2.14 (1.06)
Medium	1492 (51.2%)	346 (57%)	768 (52.3%)	812 (51.9%)	1.75 (1.05)
High	417 (16.6%)	109 (18%)	218 (17%)	235 (17.3%)	1.75 (1.08)
Smoking status					
Never	1386 (45.2%)	295 (48.5%)	685 (46.3%)	727 (46.3%)	1.83 (1.05)
Former	1160 (36.2%)	231 (35.6%)	541 (35%)	573 (34.8%)	1.85 (1.05)
Current	608 (18.6%)	113 (15.9%)	306 (18.7%)	327 (18.9%)	2.03 (1.09)
Total Diet Quality score^**^	0.67 [0.02, 1.42]	0.68 [0.03, 1.43]	0.69 [0.03, 1.43]	0.69 [0.04, 1.43]	−0.02
Sleep duration					
<6.5 HR	1477 (41.9%)	238 (32.7%)	706 (41.4%)	740 (40.6%)	2.08 (1.05)
6.5–<8.0 HR	881 (32.4%)	221 (41%)	443 (31.5%)	473 (31.7%)	1.61 (1.06)
≥8.0 HR	795 (25.7%)	181 (26.3%)	383 (27.2%)	414 (27.7%)	1.89 (1.06)
HOMA-IR^*^	1.86 (1.05)	1.27 (1.05)	1.86 (1.04)	2.68 (0.12)	NA
Family history of diabetes	1686 (46.5%)	276 (36.8%)	751 (45.7%)	792 (45.2)	2.09 (1.05)

^*∗*^Geometric means are presented for categorical variables and weighted Pearson's correlations are presented for continuous variables. Insulin resistance was collected on subjects with fasting glucose.

^**^Median [interquartile range] presented for skewed predictors.

^***^Conducted among *N* = 2175 nondiabetic subjects.

**Table 2:** Associations (Beta estimate [95% CI]) between diet quality, sleep duration, physical activity and waist circumference and insulin resistance, by socioeconomic status level.

	Education level				Income		
	Beta estimate [95% CI]^A^				Beta estimate [95% CI]^A^		
	*N* = 2359^*^				*N* = 2359^*^		
	<HS *N* = 403	HS or equiv. *N* = 698	College or equiv . *N* = 509	Postgrad *N* = 750	<$20,000 *N* = 939	$20,000–$49,999 *N* = 682	$50,000+ *N* = 738
**Individual factor**							
Model 1^C^: Total diet quality (lower tertile versus upper and middle tertiles)	0.04 [−0.13, 0.22]	0.01 [−0.12, 0.14]	0.08 [−0.04, 0.20]	−0.01 [−0.11, 0.08]	0.002 [−0.13, 0.14]	0.03 [−0.08, 0.13]	0.02 [−0.07, 0.11]
Model 2: BMI (≥25 versus <25)	**0.22** [**0.04, 0.40**]^B^	**0.23** [**0.10, 0.35**]^B^	**0.24** [**0.10, 0.38**]^B^	**0.19** [**0.11, 0.28**]^B^	**0.25** [**0.13, 0.37**]^B^	**0.22** [**0.12, 0.33**]^B^	**0.19** [**0.10, 0.28**]^B^
Model 3: Waist circumference, cm mean SE (Larger: ≥88 for women, ≥102 for men, versus smaller)	**0.33** [**0.19, 0.47**]^B^	**0.28** [**0.19, 0.37**]^B^	**0.27** [**0.16, 0.39**]^B^	**0.22** [**0.14, 0.30**]^B^	**0.27** [**0.15, 0.38**]^B^	**0.26** [**0.17, 0.36**]^B^	**0.23** [**0.15, 0.30**]^B^
Model 4: Physical activity (low versus med/high)	0.13 [−0.02, 0.27]	0.10 [−0.00, 0.21]	−0.01 [−0.14, 0.12]	0.05 [−0.03, 0.14]	0.02 [−0.08, 0.13]	0.05 [−0.04, 0.15]	0.05 [−0.04, 0.14]
Model 5: Sleep length/quality (low versus med/high)	0.05 [−0.12, 0.22]	0.05 [−0.06, 0.15]	0.03 [−0.09, 0.14]	0.03 [−0.05, 0.11]	0.06 [−0.12, 0.24]	0.04 [−0.09, 0.17]	0.01 [−0.14, 0.15]
**Two factors**							
Low diet and sleep scores	0.05 [−0.18, 0.28]	0.04 [−0.13, 0.22]	0.07 [−0.12, 0.25]	−0.03 [−0.17, 0.12]	0.07 [−0.15, 0.29]	0.16 [−0.09, 0.41]	0.18 [−0.09, 0.46]
**Three factors**							
Low diet and sleep scores, plus low activity	0.12 [−0.13, 0.36]	0.11 [−0.13, 0.34]	0.18 [−0.06, 0.42]	0.05 [−0.22, 0.32]	0.07 [−0.15, 0.29]	0.16 [−0.09, 0.41]	0.18 [−0.09, 0.46]
**Four factors**							
Low diet, sleep, activity, plus high BMI or waist (select the relevant one)	0.13 [−0.13, 0.38]	0.11 [−0.12, 0.35]	0.22 [−0.05, 0.49]	0.11 [−0.18, 0.41]	0.09 [−0.15, 0.32]	0.19 [−0.08, 0.45]	0.23 [−0.07, 0.52]

^*∗*^These analyses exclude subjects that did not have fasting glucose available. The column totals do not add up due to rounding from multiple imputations.

^A^From multivariable models adjusting for age, gender, race/ethnicity, smoking, and family history of diabetes.

^B^In bold, *P* ≤ 0.05.

^C^Dietary models are adjusted for age, gender, race/ethnicity, total caloric intake, smoking, and family history of diabetes.

**Table 3:** Association (OR [95% CI]) between diet quality, sleep duration, and physical activity and prediabetes (versus unaffected), by socioeconomic status level.

	Education level				Income		
	Odds ratio [95% CI]^A^				Odds ratio [95% CI]^A^		
	*N* = 2175^*∗*^				*N* = 2175^*∗*^		
	<HS	HS or equiv.	College or equiv.	Postgrad	<$20,000	$20,000–$49,999	$50,000+
	*N* = 323	*N* = 628	*N* = 472	*N* = 752	*N* = 782	*N* = 654	*N* = 738
**Individual factor**							
Model 1^C^: Total diet quality (lower tertile versus upper and middle tertiles)	1.57 [0.29, 8.38]	0.98 [0.40, 2.42]	1.40 [0.63, 3.12]	0.81 [0.46, 1.42]	1.06 [0.45, 2.50]	1.07 [0.54, 2.14]	0.94 [0.55, 1.61]
Model 2: BMI (≥25 versus <25)	2.08 [0.61, 7.06]	1.17 [0.53, 2.62]	1.31 [0.60, 2.87]	1.17 [0.73, 1.88]	1.08 [0.55, 2.13]	1.46 [0.75, 2.83]	1.23 [0.73, 2.07]
Model 3: Waist circumference, cm mean SE (Larger: ≥88 for women, ≥102 for men, versus smaller)	1.31 [0.47, 3.70]	1.74 [0.79, 3.86]	1.90 [0.99, 3.65]	**1.68** [**1.07, 2.62**]^B^	0.97 [0.45, 2.10]	**2.13** [**1.10, 4.10**]	**1.88** [**1.22, 2.92**]
Model 4: Physical activity (low versus med/high)	2.37 [0.70, 8.01]	1.29 [0.62, 2.66]	0.93 [0.44, 1.98]	1.09 [0.62, 1.91]	1.41 [0.67, 2.97]	1.20 [0.57, 2.53]	1.01 [0.59, 1.73]
Model 5: Sleep length/quality (low versus med/high)	2.36 [0.87, 6.40]	1.00 [0.50, 2.00]	1.20 [0.59, 2.43]	1.19 [0.74, 1.91]	1.12 [0.60, 2.10]	0.93 [0.49, 1.77]	1.45 [0.89, 2.35]
**Two factors**							
Low diet and sleep scores	3.43 [0.49, 24.18]	0.60 [0.19, 1.96]	1.13 [0.36, 3.50]	0.94 [0.36, 2.47]	0.81 [0.23, 2.87]	1.47 [0.48, 4.47]	0.92 [0.37, 2.28]
**Three factors**							
Low diet and sleep scores, plus low activity	2.83 [0.24, 33.06]	1.64 [0.37, 7.21]	1.06 [0.17, 6.48]	2.80 [0.34, 22.91]	1.43 [0.35, 5.77]	2.75 [0.28, 27.34]	2.19 [0.36, 13.38]
**Four factors**							
Low diet, sleep, activity, plus high BMI or waist (select the relevant one)	3.33 [0.29, 38.89]	1.58 [0.32, 7.74]	0.89 [0.09, 8.43]	2.02 [0.22, 18.66]	1.33 [0.33, 5.43]	2.33 [0.21, 25.44]	2.33 [0.25, 21.90]

^*∗*^These analyses exclude 980 subjects with diabetes.

^A^From multivariable models adjusting for age, gender, race/ethnicity, smoking status, and family history of diabetes.

^B^In bold, *P* ≤ 0.05.

^C^Dietary models are adjusted for age, gender, race/ethnicity, total caloric intake, and family history of diabetes.
